# Multi-Sensor monitoring dataset for milling process with varied parameters and materials

**DOI:** 10.1016/j.dib.2024.110703

**Published:** 2024-07-02

**Authors:** Guochao Li, Hao Zheng, Shixian Xu, Kunpeng Zhu, Yinfei Liu, Ru Jiang, Li Sun, Yikai Ning

**Affiliations:** a School of Mechanical Engineering Jiangsu University of Science and Technology, Zhenjiang 212100, China; b Institute of Advanced Manufacturing Technology, Hefei Institutes of Physical Science, Chinese Academy of Science, Huihong Building, Changwu Middle Road 801, Changzhou 213164, Jiangsu, China; c Department of Control Engineering, University of Rocket Force Engineering, Xi'an 710025, China

**Keywords:** Multi-sensor monitoring, Milling process, Physics Informed machine learning

## Abstract

Real-time monitoring of milling parameters is essential to improve machining efficiency and quality, especially for the workpieces with complex geometry. Its main task is to build the relationship between the parameters and the monitoring data. As the relationship is challenging to be established solely through mechanism-driven or data-driven methods, the physics informed method, based on prior physical laws between physical signals and milling parameters, becomes the optimal method. However, this method is limited due to the lack of a high-quality dataset. Therefore, a multi-sensor monitoring dataset for the milling process with various milling parameters and milling materials is built. The variables include cutting depth, cutting width, feed rate, spindle speed and workpiece materials (aluminium alloy 7030 and CK45 steel). The multi-sensor includes force, vibration, noise, and current. A dataset comprising 115 samples is built, including 100 samples collected using the 'all factors' method, and 15 slot milling samples using two different workpiece materials. The 15 slot milling samples are used to calibrate mechanical milling force coefficients, which is beneficial for developing a physics-informed machine learning algorithm.

Specifications Table

**Table:** 

Subject	Mechanical Engineering
Specific subject area	Multi-Sensor monitoring for milling process with varied milling parameters and milling materials
Data format	Multivariate time series signal, with unified coordinates and stationary signal interception
Type of data	.xlsx (Microsoft Office EXCEL File)
Data collection	Milling experiment was conducted with the 3-axis CNC milling machine. The workpieces were aluminum alloy 7030 and CK45 steel. One Solid Carbide End Mill is used to carried 100 times side milling and 15 times slot milling. The variate includes cutting depth, cutting width, feed rate, spindle speed and workpiece materials. The milling forces, vibration, noise and current were recorded with Kistler 9129AA dynamometer, Kistler 9129AA dynamometer, GRAS 46AE noise sensor, and CPL8100B low frequency current probe.
Data source location	Recorded and stored at the Institute of School of Mechanical Engineering, Jiangsu University of Science and Technology, Zhenjiang, Jiangsu Province, China
Data accessibility	Repository name: Zenodo (https://www.zenodo.org),Direct URL to data: https://zenodo.org/records/10613521 Repository name: OPENICPSR (https://www.openicpsr.org), Direct URL to data: https://doi.org/10.3886/E204021V1 Repository name: Mendely Data (https://data.mendeley.com/), Direct URL to data: https://doi.org/10.17632/8jk3kyxmbn.1

## 1 Value of the Data

• The data are valuable as they are the first to provide the multi-sensor monitoring data of milling process with various milling parameters, as well as the data for calibrating mechanical milling force coefficients. The data collected in this experiment has high accuracy and a wide variety of sensors. In the data that is currently publicly available, there are few types of sensors, and the collected signals are tool life cycle signals under one or several working conditions. Only monitoring data about tool wear were provided, and monitoring data of different milling parameters had not been reported.
• The data are helpful for developing the physics-informed machine learning algorithm for milling process monitoring. The currently publicly available dataset is based on the collection of tool wear values after each cutting process using equipment such as microscopes. The errors caused by manual measurement and data estimation can reduce the calibration accuracy of the physical model. Unlike this, the milling parameters in the dataset of this paper are directly obtained through the machine tool system, which has the characteristics of high accuracy and low error.
• The data can be used to develop new milling force equivalent models with accessible signals, such as current, vibration, and noise. It is a significant work to enable milling process monitoring method be used in practical, as the acquisition of milling force data is inconvenient, costly and time-consuming.
• The data can be used to develop milling parameters monitoring methods for complex-shaped parts milling process, whose cutting depth, cutting width, spindle speed and feed rate were always changed [1].
• The data can provide abundant research resources for scholars who are interested for mechanism model of milling force, such as the 15-slot milling experimental data that can be applied to calibrate cutting force coefficient which is essential for the milling force model [2]. However, most of the public data have no corresponding experimental data or insufficient data to calibrate the coefficient precisely.
• The amount of data provided is large and the quality of data is high because the sensors used in the experiment have multi-type, high-precision and high sampling frequency.

## 2 Data Description

The dataset consists of 115 samples. Among them, there are four sets of datasets based on the variables of cutting depth (*a_p_*) and cutting width (*a_e_*). Each set contains 25 samples. Additionally, there are 15 samples for feed slot milling experiments conducted on aluminum alloy and stainless-steel materials. The samples contain time-series data for force, vibration, noise, and current signals. Representations of the signals are shown in Table 1. Among them, Fx, Fy, and Fz represents the force signal in the X, Y, and Z direction, Vx, Vy, and Vz represent vibration signal of the spindle in the X, Y, and Z direction, SN represents the noise signal, and I_U_, I_V_, and I_W_ are the phase currents of the U, V, and W of the machine tool spindle during the machining process.

**Table 1:** Description of the signals.

Signal	Symbol
X-direction force signal (N)	Fx
Y-direction force signal (N)	Fy
Z-direction force signal (N)	Fz
Three-axis vibration signal of the spindle (g)	Vx, Vy, Vz
Noise signal (Pa)	SN
Current signal(A)	I_U_, I_V_, I_W_

The data obtained from each sample is stored in Excel files. Unstable signals during the cutting-in and cutting-out stages are removed, and 50,000 data points corresponding to stable cutting process are retained. The force, vibration, noise, and current signals under the same cutting conditions are merged into a unified file. The structure of the Excel file is as follows: the first column represents the X-direction force signal, the second column represents the Y-direction force signal, the third column represents the Z-direction force signal, the fourth to sixth columns represent the spindle X, Y, and Z-direction vibration signals, the seventh column represents the noise signal, and the eighth to tenth columns represent the current signals.

For the 100 single-factor samples, they are evenly divided into 4 groups based on spindle speed, feed rate, and workpiece material. The Excel file naming format is: Gx_xx, where Gx represents the x-th group, and xx represents the xx-th sampling within the x-th group. For example, G1_01 represents the first sampling of Group 1. The data structure for storing data is shown in Table 2.

**Table 2:** Data storage sample.

No.	Fx	Fy	Fz	Vx	Vy	Vz	SN	I_U_	I_V_	I_W_
1	12.2	25.9	-5.6	-0.9	-0.6	0.4	-0.1	-6.8	7.5	-0.9
2	12.7	25.6	-3.8	-0.8	0.5	-0.4	-0.1	-7.1	7.1	-0.2
⋮	⋮									
50,000	21.4	43.6	-18.3	-0.3	-0.3	-1.4	-0.4	4.5	4.6	-8.8

As for the feed slot milling samples, they are divided into 2 groups based on workpiece material. The data format is the same as that of the single-factor samples. The Excel file naming format is as follows: M_7030_x, M_45_x.

The collected force signals are the force acting on the workpiece. The direction of the vibration signal depends on the orientation of the vibration sensor. The coordinate axes of these two signals are not uniform. To achieve consistency in expression and facilitate potential research, the coordinate axes of these two signals are unified to the XYZ coordinate system. The corresponding relationships of each coordinate are presented in Table 3. Among them, X_D_Y_D_Z_D_ are the coordinates of dynamometer, and X_V_Y_V_Z_V_ are the coordinates of the three-axis vibration sensor.

**Table 3:** Signal coordinate comparison.

Unified coordinates	X	Y	Z
Vibration signal coordinates	Y_V_	-X_V_	-Z_V_
Dynamometer coordinates	X_D_	-Y_D_	Z_D_

After unifying the coordinate system for cutting force and vibration, the final expressions for force signal and vibration signal are as follows:

(1) $$\left\{\begin{array}{cc} F_{x} & V_{x}\\ F_{y} & V_{y}\\ F_{z} & V_{z} \end{array}\right\}=\left\{-\begin{array}{cc} F_{xD} & V_{yV}\\ F_{yD} & -V_{xV}\\ F_{zD} & -V_{zV} \end{array}\right\}$$

where F_xD_, F_yD_, and F_zD_ represent the forces in the coordinates of dynamometer in the X_D_, Y_D_, and Z_D_ directions. V_xV_, V_yV_, and V_zV_ represent the vibration signals in the X_V_, Y_V_, and Z_V_ directions in the coordinate system of the triaxial vibration sensor.

After unifying the coordinate system, it is necessary to intercept effective signals. Taking the X-direction force signal of sample G1_ 01 as an example, in the initial stage, the signal amplitude continuous increase when the tool cuts into the workpiece, As the cutting stabilizes, the signal also stabilizes. At the end, when the tool cuts out, the signal amplitude decreases until it reaches zero. The entire signal change conforms to the actual cutting process. In the practical application of data, the signal of the feed and retreat process cannot effectively reflect the actual working conditions in the machining process. Therefore, the first and last signals are truncated, and 50,000 data points in the stable stage are retained.

**Fig. 1:**
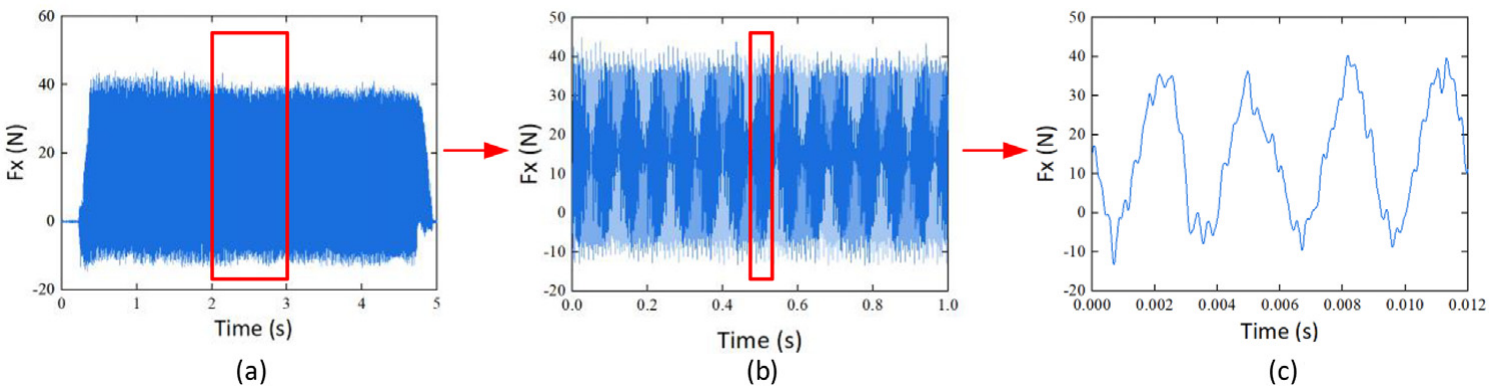
X-direction milling force signal processing process. (a) The overall signal. (b) The signal with 50,000 data points extracted. (c) The signal rotated for one revolution.

Taking the X-direction force signal of sample G1_01 as an example, the data process procedure includes the overall signal, the signal with 50,000 data points extracted, and the signal rotated for one revolution. In this scenario, the spindle speed is 5000 rev/min, and due to a sampling frequency of 50 kHz, 600 data points are collected for one complete rotation of the tool. The detailed processing procedure is illustrated in Fig. 1.

**Fig. 2:**
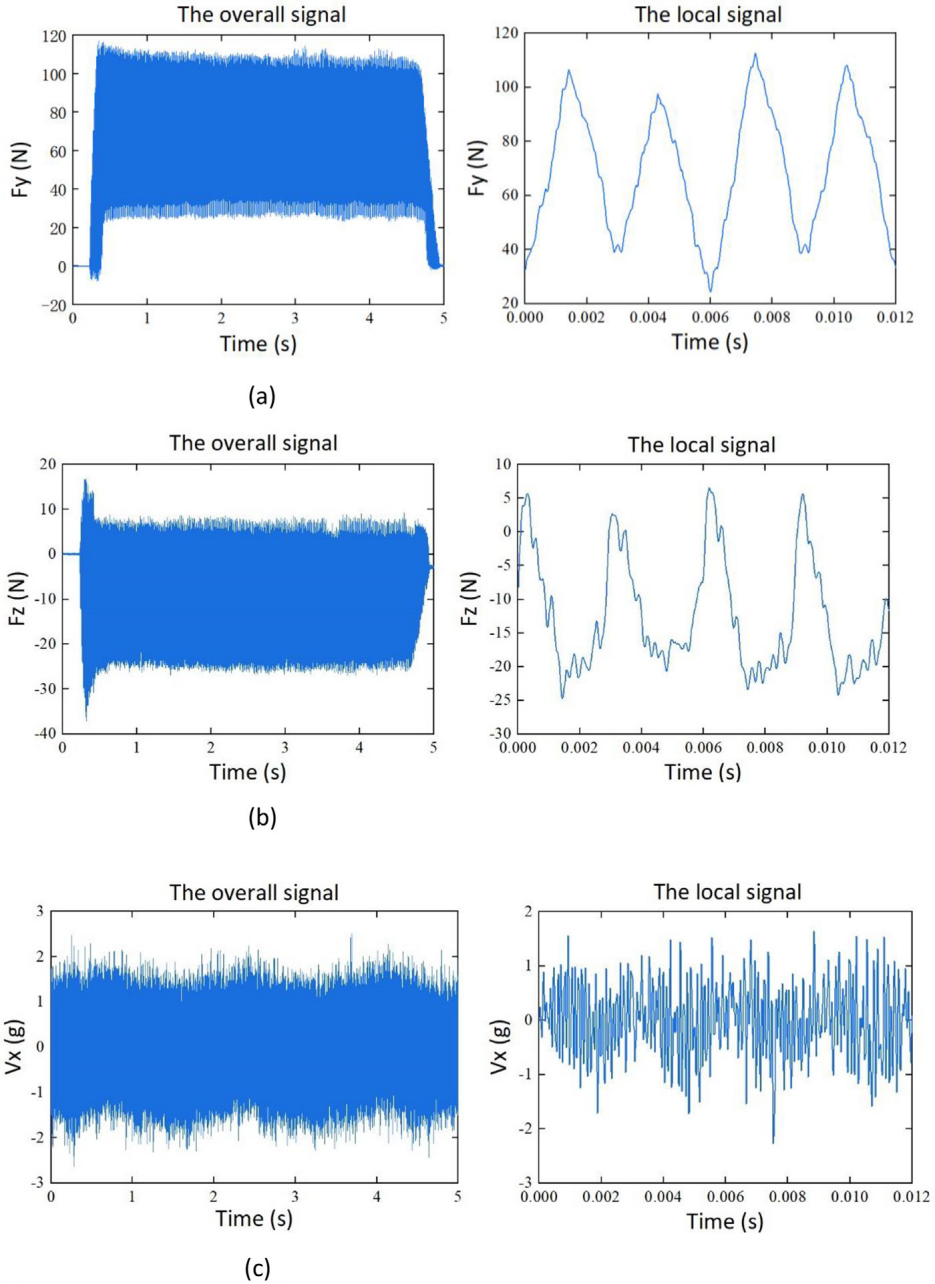

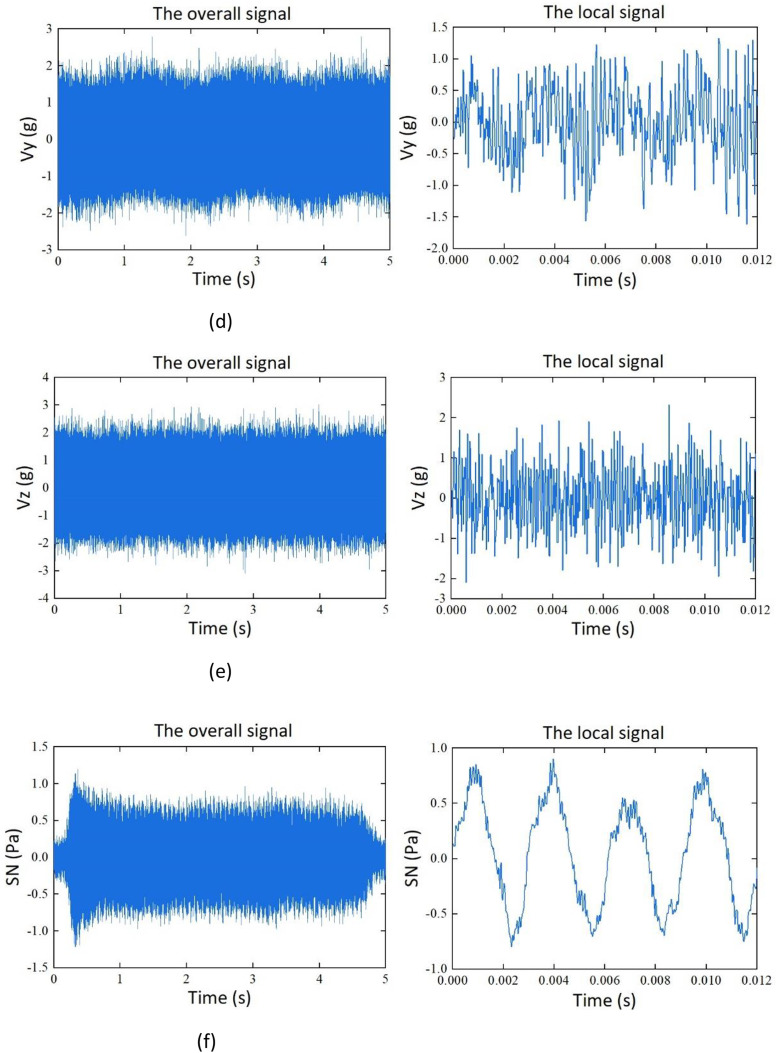

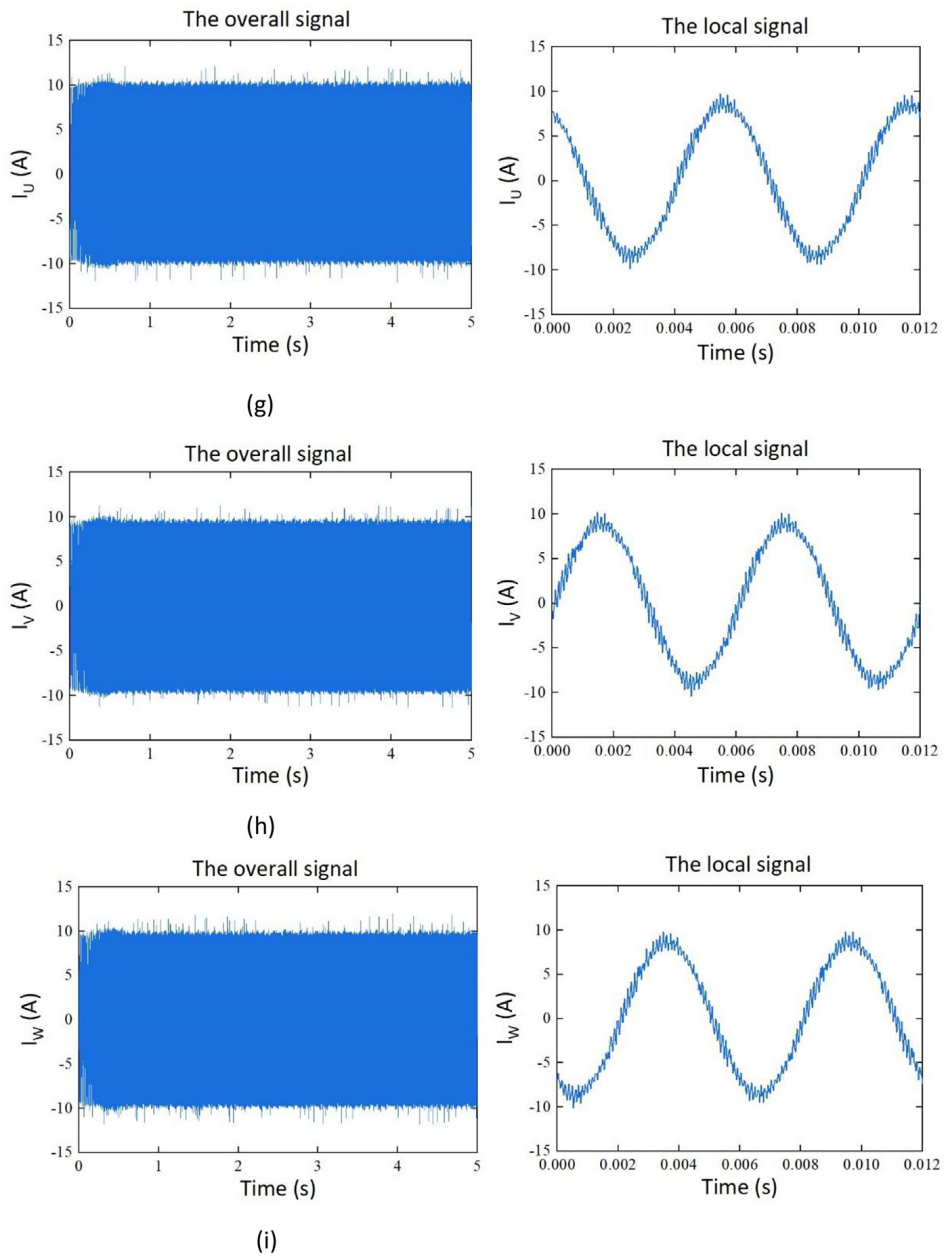
Signals of cutting force, vibration, noise, and current. (a) Y-direction milling force signal. (b) Z-direction milling force signal. (c) X-direction vibration signal. (d) Y-direction vibration signal. (e) Z-direction vibration signal. (f) Noise signal. (g) current signal I_U._ (h) current signal I_V._ (i) current signal I_W_.

Taking sample G1_01 as an example, the signals of cutting force, vibration, noise, and current in the unified coordinate system, are shown in Fig. 2.

## 3 Experimental Design, Materials and Methods

The milling parameters of 4 groups were presented in Table 4. The cutting depth (*a_p_*) and the cutting width (*a_e_*) are variables for each group. The values of a_p_ and a_e_ are set as [2, 1.8, 1.6, 1.4, 1.2] according to the tool radius and dry cutting conditions, because higher material removal rates can lead to disasters such as machining chatter and tool abnormality while lower material removal rates may result in lower signal-to-noise ratio. Each group constitutes a full factorial design with two factors and five variables. The constant milling parameters for group 1 include the spindle speed of 5000 rev/min, the feed rate of 800 mm/min, and aluminum alloy 7030 workpiece material.

**Table 4:** The milling parameters for the experiment.

No.	*a_p_* (mm)	*a_e_* (mm)	Spindle speed (rev/min)	Feed rate (mm/min)	Workpiece material
G1_1	2	2	5000	800	aluminum alloy 7030
G1_2		1.8			
G1_3		1.6			
G1_4		1.4			
G1_5		1.2			
G1_6	1.8	2			
G1_7		1.8			
G1_8		1.6			
G1_9		1.4			
G1_10		1.2			
G1_11	1.6	2			
G1_12		1.8			
G1_13		1.6			
G1_14		1.4			
G1_15		1.2			
G1_16	1.4	2			
G1_17		1.8			
G1_18		1.6			
G1_19		1.4			
G1_20		1.2			
G1_21	1.2	2			
G1_22		1.8			
G1_23		1.6			
G1_24		1.4			
G1_25		1.2			
G2_1-G2_25	Same as G1	Same as G1	10,000	800	aluminum alloy 7030
G3_1-G3_25	Same as G1	Same as G1	5000	1600	aluminum alloy 7030
G4_1-G4_25	Same as G1	Same as G1	5000	800	CK45 steel

**Fig. 3:**
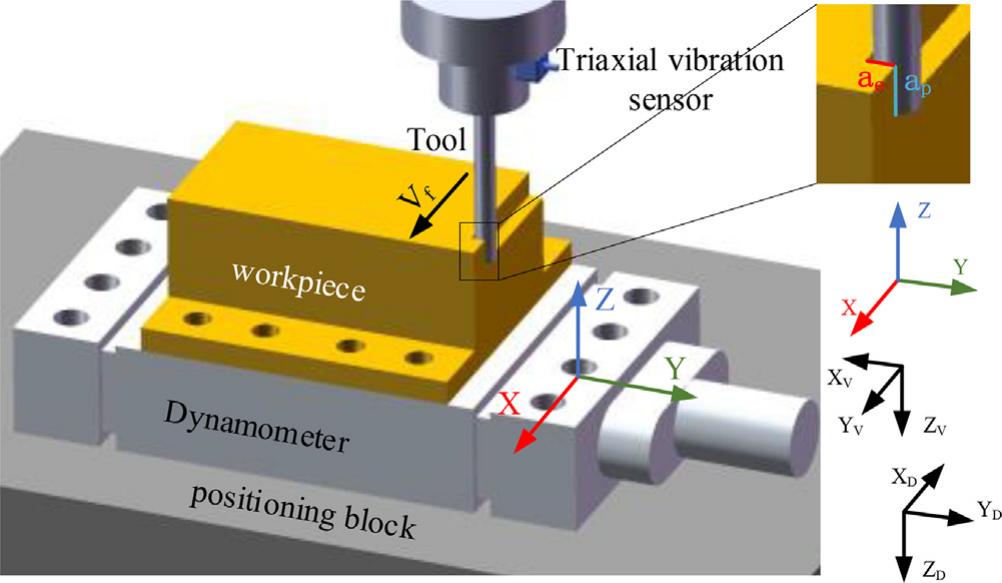
Schematic diagram of milling machining.

**Fig. 4:**
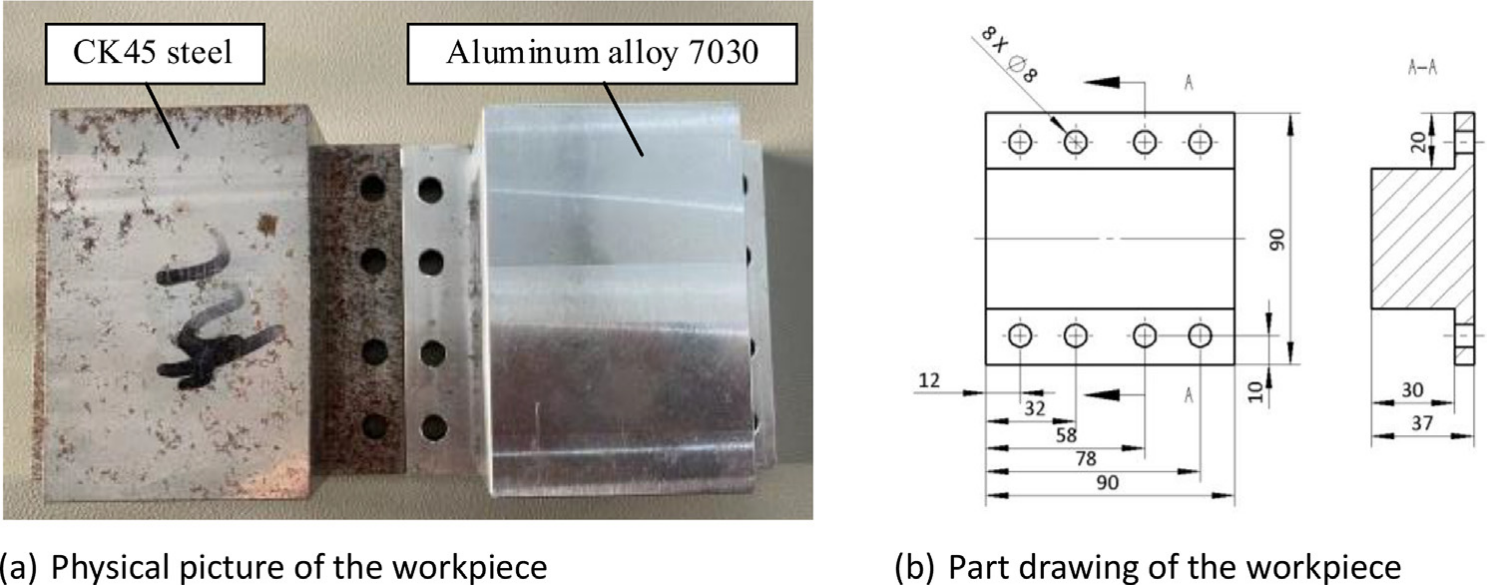
Experiment workpiece.

Additionally, single-factor experiments are designed for group 2–4 with spindle speed, feed rate, and workpiece material as variables. The spindle speed is changed to be 10,000 rev/min for group 2 and feed rate is set to be 1600 mm/min for group 3. Accordingly, CK45 steel instead of aluminum alloy 7030 is used as the workpiece material for group 4. The spindle speeds of 5000 and 10,000 rev/min, as well as feed speeds of 800 and 1600 mm/min, are selected for the experiment based on the workpiece material, tool strength, and previously determined a_p_ and a_e_ values. The above samples have the same cutting path, which is straight lines along the X direction, as shown in Fig. 3. The workpiece is a cuboid, including two different materials: aluminum alloy 7030 and CK45 steel. The reason for choosing these two materials is that both are widely used materials [3,4]. In addition, there are certain differences in their physical properties, which can reflect the influence of materials on signals during the processing. The workpiece dimensions are shown in Fig. 4.

Cutting force is considered to be the most effective physical quantity to reflect the machining mechanism in milling process [5]. The calibration of cutting force coefficient is one of the key steps in modeling cutting forces which is usually achieved through the method of average cutting force calibration [6]. To obtain the average cutting force within one full revolution of the tool, 15 slot milling experiments were designed. The milling parameters are presented in Table 5.

**Table 5:** The milling parameters for 15 slot milling experiments.

Workpiece material	Spindle speed (rev/min)	*a_p_* (mm)	*a_e_* (mm)	Feed per tooth (mm/tooth)
Aluminum alloy 7030	5000	1	6	0.020, 0.030, 0.040, 0.050, 0.060, 0.070, 0.080
CK45 steel	5000	1	6	0.020, 0.025, 0.030, 0.035, 0.040, 0.045, 0.050, 0.055

**Fig. 5:**
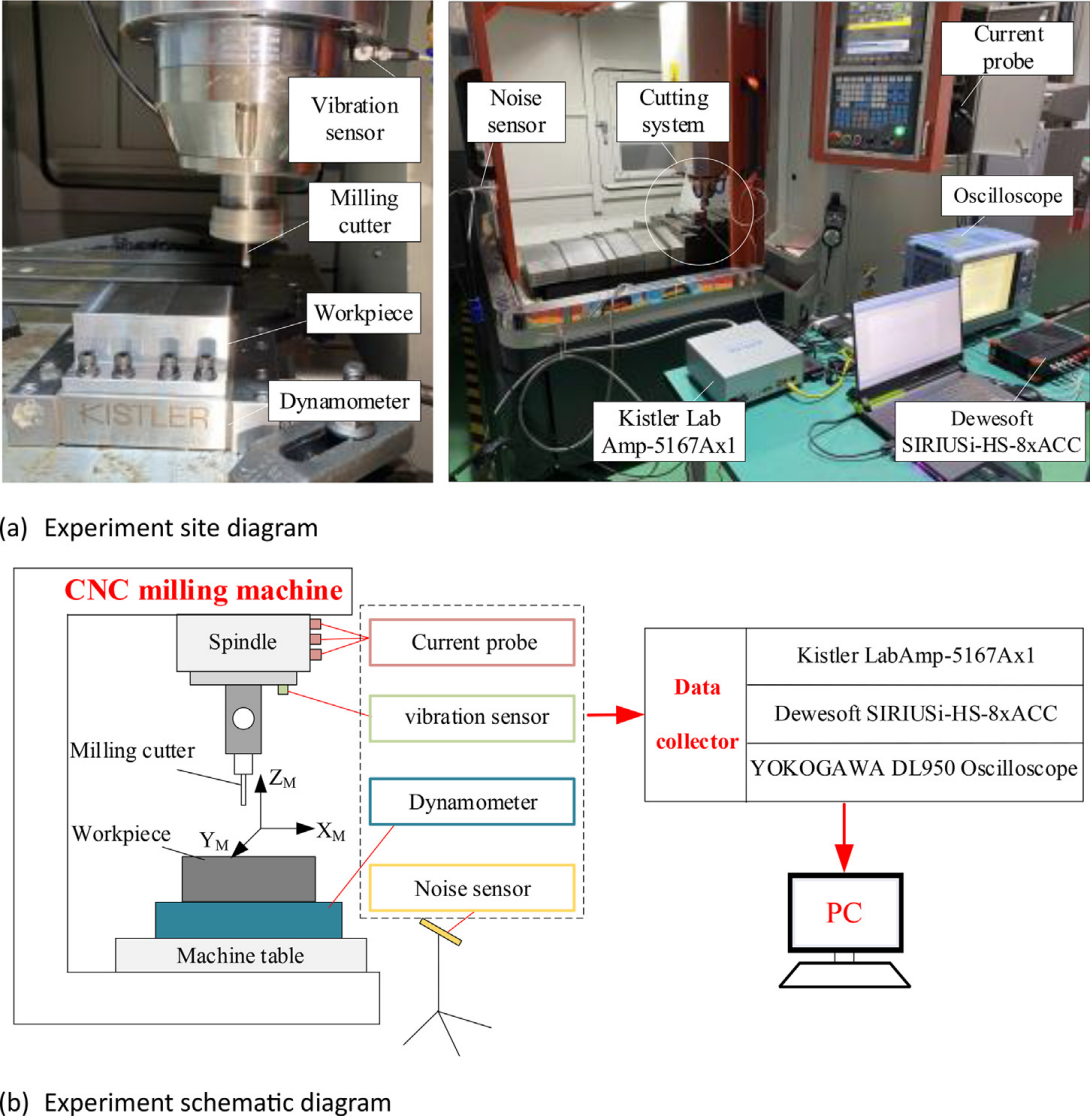
Milling experiment system.

**Fig. 6:**
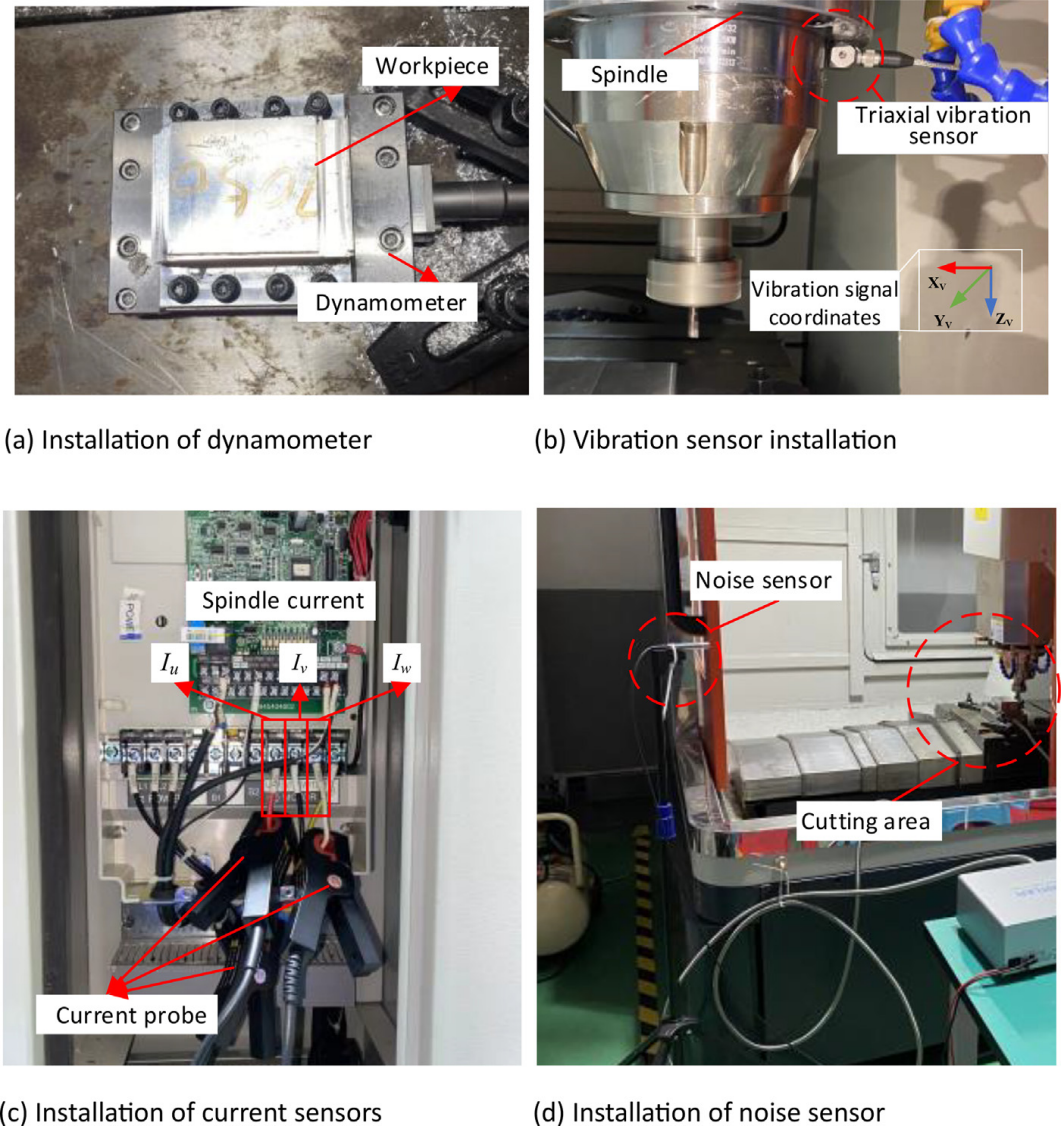
Sensor installation.

Four types of the physical signals, force, vibration, noise and current, were collected during the cutting process. The experimental equipment and sensors are shown in Fig. 5. A triaxial vibration sensor was installed on the spindle of the DX-650 high-speed CNC milling machine. The force sensor was mounted on a positioning block and the workpiece was securely attached to the force sensor using bolts. The noise sensor was affixed to a tripod near the worktable of the machine tool. The current probe was clamped onto the electrical line of the spindle for current signal acquisition. The installation location of the sensor is shown in Fig. 6.

**Fig. 7:**
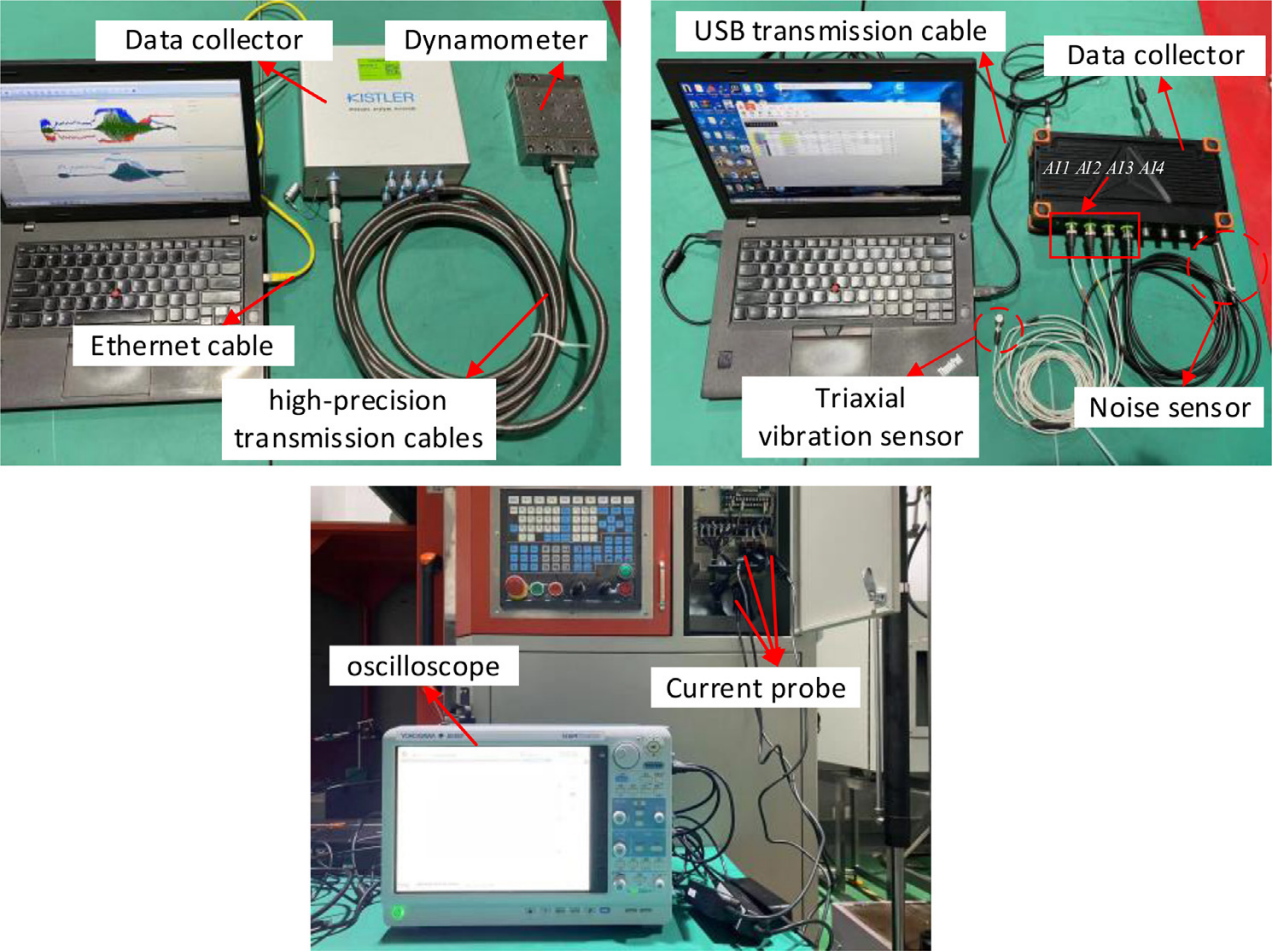
Interconnection of signal acquisition systems.

After sensor installation, it is necessary to interconnect the sensor, data collector, and PC to form a complete processing and testing platform. The triaxial vibration sensor and the noise sensor are connected to the Dewesoft SIRIUSi-HS-8xACC data collector unit via communication cables. The X Y Z axes of the vibration sensor and the noise sensor are connected to channels *AI1, AI2, AI3*, and *AI4* of the data acquisition unit, respectively. The data collector unit is then connected to PC via a USB transmission cable. The Kistler 9129AA force sensor is connected to the Kistler LabAmp-5167Ax1 data acquisition unit using high-precision transmission cables, and the data collector unit is connected to PC via an Ethernet cable. The CPL8100B low-frequency current probe is directly connected to the DL950 oscilloscope. The equipment connections are illustrated in Fig. 7. The experimental design involved milling operations on two different workpiece materials using a single tool. The experimental conditions are detailed in Table 6.

**Table 6:** Experiment conditions for milling machining.

Parameter	Model and value
CNC	DX-650 high-speed CNC milling machine
Tool	SECO four tooth hard alloy end milling cutter (helix angle 48°,Φ6)
Sensor	Kistler 8763B triaxial vibration sensor, CPL8100B low frequency current probe, GRAS 46AE noise sensor, Kistler 9129AA dynamometer
Data collector	Dewesoft SIRIUSi-HS-8xACC, YOKOGAWA DL950 oscilloscope, Kistler LabAmp-5167Ax1
Cooling conditions	Dry cutting
Milling mode	Down milling
Sampling frequency	50kHz

The experiments were single-factor trials with cutting depth (*a_p_*) and cutting width (*a_e_*) as variables. There were five values for both a_p_ and a_e_, resulting in a total of 25 trials. Subsequently, single-factor trials were repeated with variations in spindle speed, feed rate, and workpiece material. Each trial collected cutting force, vibration, noise, and current signals during the machining process. In addition, average milling force experiments were conducted for two different workpiece materials using slot milling. The comprehensive research methods enable this dataset to have a wider range of application scenarios. The difference in application scenarios between the currently publicly data [[7], [8], [9], [10]] and our “JUST_CAS_milling” data is listed in Table 7.

**Table 7:** Applicable scope of milling datasets.

Application Scenario	NASA [7]	PHM [8]	SDU-QIT [9]	NUAA [10]	JUST_CAS_milling
1. Training of residual life prediction model	√	√	√	√	
2. Training of a life prediction model for multiple operating conditions	√		√	√	
3. Integral end milling cutter		√	√	√	**√**
4. Calibration of milling force coefficient					**√**
5. Training of cutting depth monitoring model				√	**√**
6. Training of cutting width monitoring model					**√**
7. Force signal		√		√	**√**
8. Vibration signal	√	√	√	√	**√**
9. Noise signal					**√**
10. Acoustic emission signal	√	√			
11. Current signal	√			√	**√**

## Limitations

All experiments were carried out with the same tool. The latter record data may be disturbed by tool wear effect.

Due to the high strength of CK45 steel, during the G4 sample collection process, stronger noises were induced in the signals, which increases the workload for noise reduction.

## Ethics Statement

The present investigation did not involve human participants, animal trials, or social media data collection.

## CRediT authorship contribution statement

**Guochao Li:** Conceptualization, Methodology, Writing – review & editing. **Hao Zheng:** Validation, Investigation, Writing – original draft. **Shixian Xu:** Visualization, Software. **Kunpeng Zhu:** Supervision. **Yinfei Liu:** Investigation, Data curation. **Ru Jiang:** Investigation, Validation. **Li Sun:** Writing – review & editing. **Yikai Ning:** Investigation, Validation.

## Data Availability

JUST_CAS_milling (Original data) (Zenodo)
